# Surgical approach to a rare case of Beckwith Wiedemann syndrome with left thigh hyperplasia

**DOI:** 10.1016/j.jpra.2023.12.004

**Published:** 2023-12-12

**Authors:** F. Gesuete, M. Molle, L. Cagiano, L. Annacontini, V. Verdura, G.F. Nicoletti, G. Ferraro, D. Parisi, A. Portincasa

**Affiliations:** a Plastic and Reconstructive Surgery Unit, Multidisciplinary Department of Clinical and Experimental Medicine. University of Foggia - Medical School of Foggia, Italy; b Plastic and Reconstructive Surgery Unit, Multidisciplinary Department of Medical-Surgical and Dental Specialties, University of Campania Luigi Vanvitelli, Napoli, Italy

**Keywords:** Beckwith-wiedemann syndrome, Vertical thigh lift, Hemihyperplasia, Psycological well being, Surgery of rare case, Body simmetrization

## Abstract

Thigh lift surgery is generally performed in patients with severe weight loss outcomes, particularly those undergoing bariatric surgery. However, there are other congenital malformation conditions that may require the same treatment, such as Beckwith Wideman syndrome.

## Background

Beckwith Wideman is a rare syndrome (1 case every 10,000 newborns)^1^ characterized by a variety of clinical forms, united by the presence of hypertrophy of some parts of the body (such as the tongue or the limbs).^2^ Some authors have in the past evaluated how the hypertrophy of some organs, such as the tongue, and its surgical correction can influence the patients' quality of life,^3^^,^^4^ but in the literature there are rare if not absent works that evaluate the impact of hypertrophy of other parts of the body, such as the thigh.

Therefore, we decided to evaluate how a thigh lift intervention (a surgical technique mainly used in the treatment of patients with rapid weight loss following bariatric surgery, with a considerable improvement of body esteem and quality of life^5^) could allow for symmetrisation of the lower limb in a patient with this syndrome and quantify how much this intervention could improve her quality of life.

## Case report

A 21-year-old patient presented to our center with a chief complaint of thigh asymmetry, which had caused her significant psychological distress. Upon reviewing her medical history, it was determined that the asymmetry had been present since she was 6 months old. The diagnostic process was initiated during this time to investigate the underlying cause, leading to the identification of hyperplasia. Throughout her life, assessments revealed abdominal visceromegaly characterized by enlarged liver, gallbladder, and spleen. However, no functional abnormalities were detected, and no symptoms other than thigh asymmetry were observed.

A previous genetic study had identified anomalies related to Beckwith-Wiedemann Syndrome on the 11p15.5 locus using an oral brush sample. However, a blood sample did not exhibit the same genetic mutation, ruling out a hereditary origin. These results, combined with the patient's clinical history and morphology, led geneticists to narrow down the diagnostic possibilities to Beckwith-Wiedemann syndrome or 11p15.5 hemihyperplasia. Unfortunately, the patient did not possess more precise documentation regarding the specific mutation, and efforts to obtain further information were unsuccessful.

**Figure 1:**
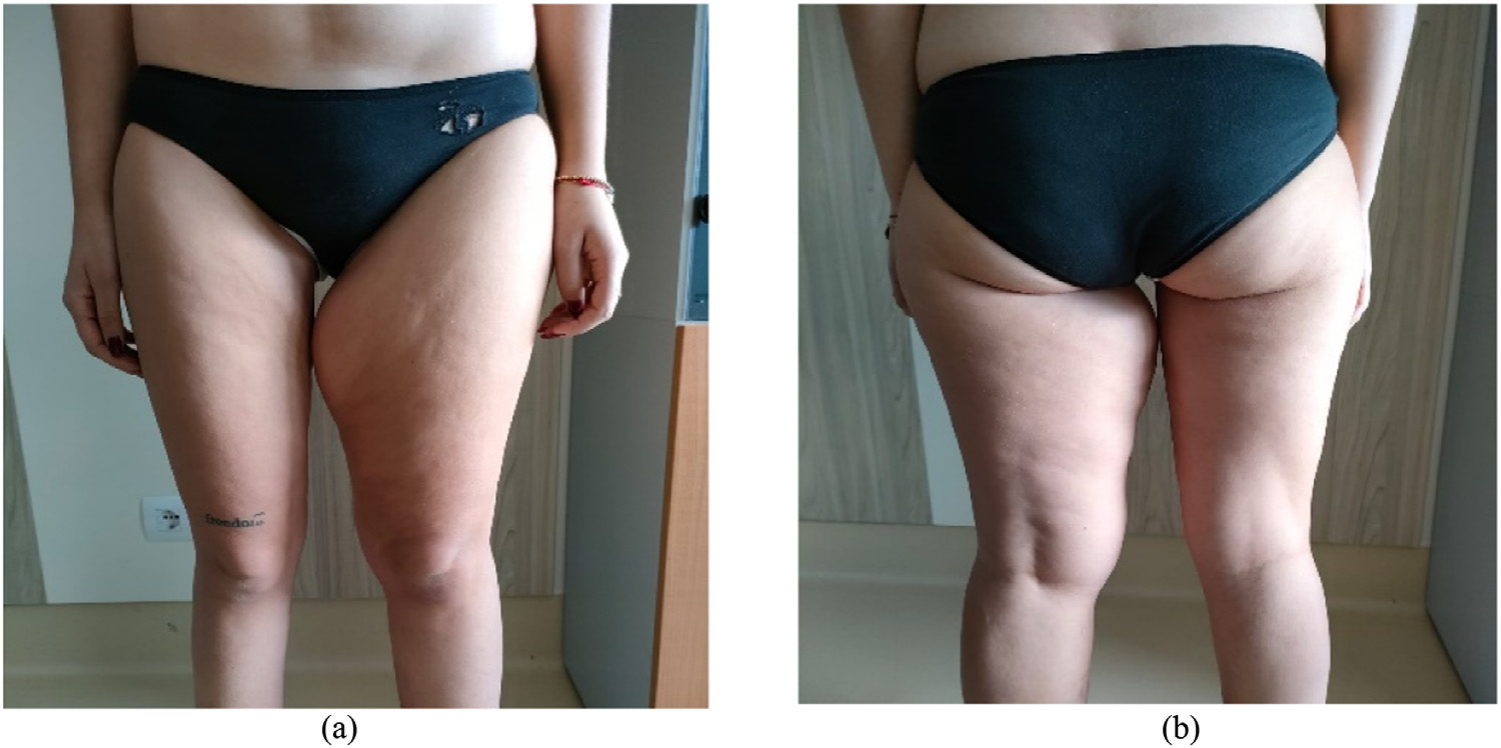
a-b: Preoperative appeareance.

During the initial interview, the patient exhibited skin and subcutaneous hypertrophy in the left thigh, specifically in the proximal medial third, accompanied by localized adiposity. Notably, the entire left lower limb displayed hypertrophy compared to the contralateral limb (Figure 1a-b). The patient reported experiencing discomfort in daily activities and significant psychological distress due to the pronounced hypertrophy on the affected side, as evidenced by her responses to the Body-QoL questionnaire^6^. Measurements revealed that the left thigh measured 62 cm, while the right thigh measured 46 cm (measured at mid-thigh level).

**Figure 2:**
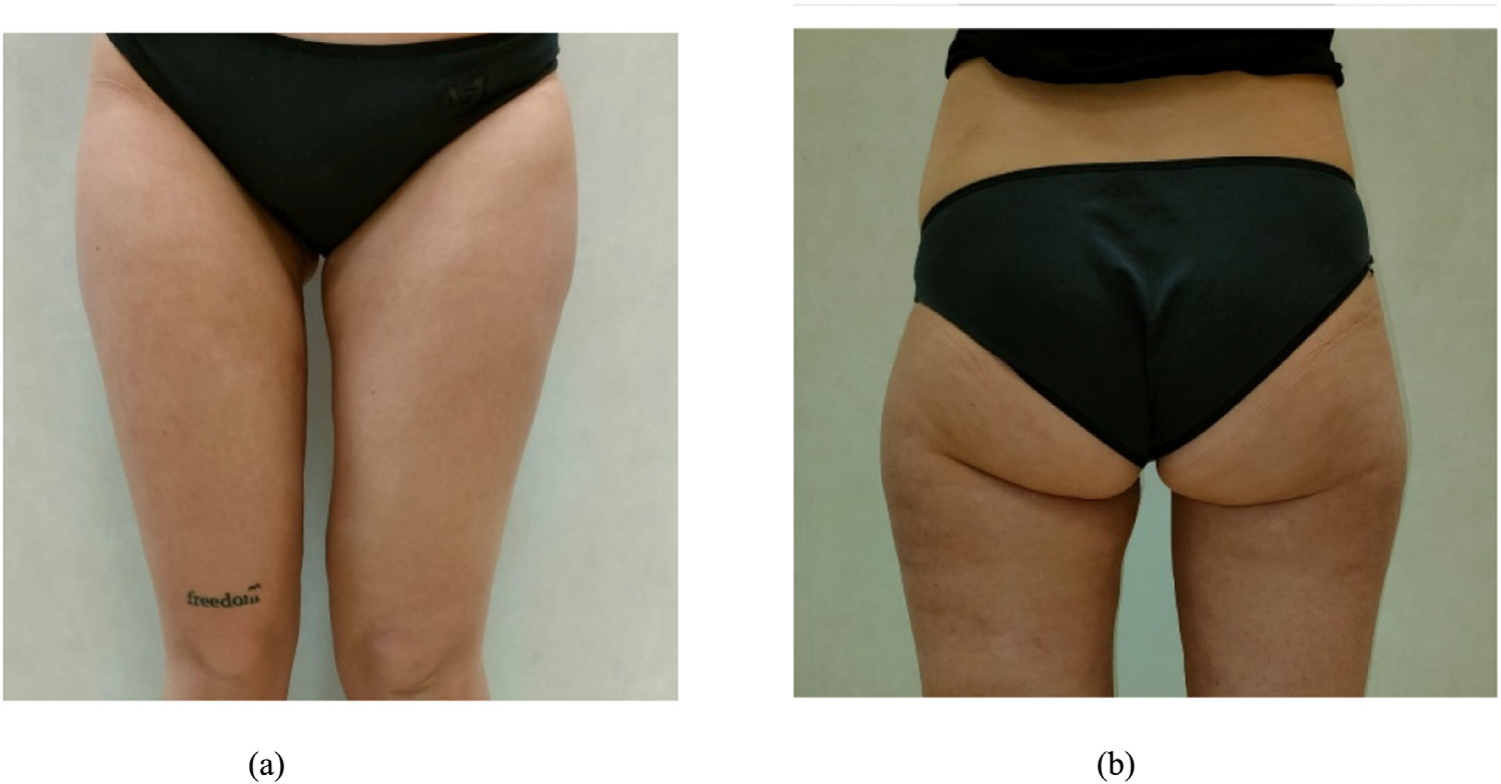
a-b: Postoperative appearance – Follow up at 6 months.

On the day of the surgery, a liposuction procedure followed by a vertical thighplasty was performed. Before the procedure, Klein's solution was administered. Liposuction was carried out, removing approximately 1300cc of adipose tissue using 4 mm cannulas for the deeper subcutaneous fat and 3 mm cannulas for the superficial fat. Subsequently, a dermo-adipose flap measuring approximately 32 × 44 cm was excised (Figure 2a-b). Remarkably, even in the immediate postoperative period, an improvement in the symmetry of both limbs was evident (Figure 2a-b).

Complications and postoperative pain were assessed using the Visual Analog Scale (VAS) at T0 (1 day), T1 (7 days), and T2 (14 days) after the surgery. Scar quality in the postoperative period was evaluated using the Vancouver Scale at T3 (1 month) and T4 (6 months).

In the postoperative period, no complications were observed, with a rapid reduction in pain (as indicated by the decrease in the VAS scale score^7^- 5 T0, 3 T1 and 1 T2) and a quick return to normal daily activities. At the 6-month follow-up, we noted a symmetrisation of the limbs (with the operated limb reducing from 62 cm to 48 cm in circumference- Figure 2a-b).

The surgical scar was found to be in excellent condition and was evaluated using the Vancouver Scar Scale,^8^ achieving a score of 2 out of 13. This indicates that the scar had minimal visibility or was well-healed.

Furthermore, the patient reported a significant enhancement in her quality of life, particularly in the domains of relationships and sexual aspects. This improvement was so profound that it led her to make the decision to pursue pregnancy. Prior to the surgery, her average score on the Body-QoL questionnaire was 12.5, which increased to 27.5 after the surgery.

## Discussion and conclusion

In our case, the patient presented with a milder form of Beckwith–Wiedemann syndrome (BWS), characterized by an excess of adipose and cutaneous tissue in the upper third of the left thigh, resulting in limited mobility and psychological discomfort. Due to the patient's modest weight loss, a combination of liposuction and dermolipectomy was necessary. We performed the procedure using the traditional technique without radiofrequency or emulsion systems. Approximately 1300 cc of fluid was aspirated, followed by the removal of the skin and subcutaneous tissue flap with ease. Minimal bleeding was observed, with the patient experiencing only a 2-point decrease in hemoglobin levels. Given the absence of significant ptosis and laxity in the proximal third of the thigh, we opted not to proceed with the classic T incision, instead performing a vertical diamond incision. The removal of the localized adiposity in the medial region allowed the patient to enhance her walking ability, which had previously been hindered by the presence of the mass. The pre-operative planning and the surgical technique made it possible to obtain a good preservation of the lymphatic network, with no oedema of the inferior limb detected in the post-operative time.

This surgical approach is commonly utilized bilaterally in the post-bariatric population, and numerous studies^5^^,^^9^,^10^ have demonstrated its effectiveness in improving quality of life to a comparable extent as seen in our patient's case. However, there remains a dearth of literature specifically focused on this particular patient population. This may be attributed to the rarity of the condition and the lack of a comprehensive approach that considers the holistic well-being of individuals with this syndrome. While significant attention is often directed towards addressing the more noticeable deformities associated with this syndrome, such as macroglossia and facial hyperplasia,^3^^,^^4^ the resolution of these conditions may leave patients with "minor" deformities that can still exert a considerable impact on their daily lives, as illustrated by our patient's experience. In an era of medicine that increasingly emphasizes the comprehensive well-being of patients, it becomes imperative to integrate more holistic approaches in the treatment of such conditions.

## Declaration of Competing Interest

The authors have no financial interest to declare in relation to the content of this article. The study was set up according to the ethical principles reported in the Helsinki declaration. The patient consented to the publication of the case.
